# Validity study of the Three-Dimensional Work Fatigue Inventory among higher education nursing faculty

**DOI:** 10.1590/0034-7167-2023-0020

**Published:** 2023-10-09

**Authors:** Évilin Diniz Gutierres Ruivo, Laurelize Pereira Rocha, Jamila Geri Tomaschewski-Barlem, Graziele de Lima Dalmolin, Laís Farias Juliano, Janaína Cassana Mello Yasin

**Affiliations:** I Universidade Federal do Rio Grande. Rio Grande, Rio Grande do Sul, Brazil; II Universidade Federal de Santa Maria. Santa Maria, Rio Grande do Sul, Brazil

**Keywords:** Faculty, Nursing, Nursing, Universities, Validation Study, Fatigue., Docentes de Enfermería, Enfermería, Universidades, Estudio de Validación, Fatiga., Docentes de Enfermagem, Enfermagem, Educação Superior, Estudo de Validação, Fadiga.

## Abstract

**Objective:**

to adapt and validate the Three-Dimensional Work Fatigue Inventory (3D-WFI) for Brazil’s cultural reality and assess the psychometric properties.

**Methods:**

a methodological study developed in six stages: initial translation; synthesis of translations; back translation; review by expert committee; pretest; and review of the adaptation process by the researchers. For validity, the instrument was applied to a sample of 318 nursing professors from Brazilian federal and state public universities. Data were analyzed using exploratory and confirmatory factor analysis, composite reliability and instrument reliability (Cronbach’s alpha and McDonald’s omega).

**Results:**

the 3D-WFI instrument showed excellent internal consistency (α=0.95 and ω=0.97), three dimensions and explained variance of 62.77%.

**Conclusions:**

the Brazilian version of the instrument showed excellent psychometric properties for assessing fatigue among Brazilian workers.

## RESUMO

**Objetivo:**

adaptar e validar o *Three-Dimensional Work Fatigue Inventory* (3D-WFI) para a realidade cultural do Brasil e avaliar as propriedades psicométricas.

**Métodos:**

estudo metodológico, desenvolvido em seis etapas: tradução inicial; síntese das traduções; retrotradução; revisão pelo comitê de especialistas; pré-teste; e revisão do processo de adaptação pelos pesquisadores. Para a validação, o instrumento foi aplicado em uma amostra de 318 indivíduos enfermeiros docentes de universidades públicas federais e estaduais brasileiras. Os dados foram analisados mediante análise fatorial exploratória e confirmatória, fidedignidade composta e confiabilidade do instrumento (alfa de Cronbach e ômega de McDonald).

**Resultados:**

o instrumento 3D-WFI apresentou excelente consistência interna (α=0,95 e ω=0,97), três dimensões e variância explicada de 62,77%.

**Conclusões:**

a versão brasileira do instrumento mostrou excelentes propriedades psicométricas para avaliação da fadiga entre trabalhadores brasileiros.

## RESUMEN

**Objetivo:**

adaptar y validar el Three-Dimensional Work Fatigue Inventory (3D-WFI) para la realidad cultural de Brasil y evaluar las propiedades psicométricas.

**Métodos:**

estudio metodológico desarrollado en seis etapas: traducción inicial; síntesis de traducciones; traducción inversa; revisión por comité de expertos; preprueba; y revisión del proceso de adaptación por parte de los investigadores. Para la validación, el instrumento se aplicó a una muestra de 318 profesores individuales de enfermería de universidades públicas federales y estatales brasileñas. Los datos se analizaron mediante análisis factorial exploratorio y confirmatorio, confiabilidad compuesta y confiabilidad del instrumento (alfa de Cronbach y omega de McDonald).

**Resultados:**

el instrumento 3D-WFI mostró excelente consistencia interna (α=0,95 y ω=0,97), tres dimensiones y varianza explicada de 62.77%.

**Conclusiones:**

la versión brasileña del instrumento mostró excelentes propiedades psicométricas para evaluar la fatiga entre trabajadores brasileños.

## INTRODUCTION

The desire to understand the causes and impact of workplace fatigue began in the early 20^th^ century. Workplace fatigue features prominently in theoretical perspectives on workers’ health^(1)^. It is seen as a personal and work-related outcome, and is also an essential construct in investigations of workers’ safety and physical and mental health that can link working conditions to employee attitudes and performance^(2)^.

The modern work environment requires more intense interpersonal interactions between workers and between them and other people outside the organization. In this regard, interest in another type of fatigue arose, such as emotional fatigue, which results from emotional energy depletion. Thus, the authors^(2)^ proposed the following definition: workplace fatigue represents extreme tiredness and reduced functional capacity, which is felt during and at the end of a working day, which involves three types of energy resources: physical (extreme physical tiredness and reduced ability to engage in physical activity); mental (extreme mental fatigue and reduced ability to engage in cognitive activities); and emotional (extreme emotional exhaustion and reduced ability to engage in emotional activity)^(2)^.

There are instruments^(3)^ that measure workplace fatigue, however these instruments assess fatigue in a punctual or unidimensional way. Measuring workplace fatigue can be challenging and a tool for measuring workplace fatigue must be multidimensional, separately assessing physical, mental and emotional workplace fatigue dimensions^(2)^.

In this context, the authors^(2)^ developed the Three-Dimensional Work Fatigue Inventory (3D-WFI), which provides a complete and proportional assessment of fatigue. The 3D-WFI is the first instrument developed to measure workplace fatigue in three dimensions. It consists of 18 items, which assess physical, mental and emotional fatigue^(2)^.

Originally, the instrument was developed in English^(2)^ and validated in different populations of workers^(1-7)^. It was identified that this is the main measure to assess workplace fatigue, translated and adapted into German^(1)^, Arabic^(4,6)^, Persian^(5)^ and Norwegian^(7)^. In all these languages, the instrument presented good psychometric evidence. However, until now, there are no instruments that assess workplace fatigue for the Brazilian population.

The 3D-WFI is an instrument that may facilitate future investigations into work fatigue as a detrimental consequence and as a potential cause of a variety of work-related attitudes, behaviors and dysfunctional outcomes. Moreover, it is noteworthy that using 3D-WFI helps nurses to reflect on workers’ health care, making it possible to subsidize actions that minimize the negative effects of workplace fatigue.

## OBJECTIVE

To adapt and validate the Three-Dimensional Work Fatigue Inventory (3D-WFI) for Brazil’s cultural reality and assess the psychometric properties.

## METHODS

### Ethical aspects

The study was conducted in accordance with national and international ethics guidelines, as was approved by the Research Ethics Committee of the *Universidade Federal do Rio Grande*, whose opinion is attached to this submission. All research participants were guaranteed data confidentiality and anonymity, and the Informed Consent Form was obtained from all individuals involved in the study online.

### Study design, place and period

This is a methodological study of cross-cultural adaptation, based on the Consensus-based Standards for the selection of health Measurement Instruments (COSMIN) guidelines. It was carried out with emphasis on the 3D-WFI scale - Brazil psychometric assessment after a careful process of cross-cultural adaptation^(8)^. Data collection was carried out in a virtual environment with national coverage in the five regions of Brazil (North, Northeast, Midwest, Southeast and South), and was carried out between July and November 2021.

### Population or sample; inclusion and exclusion criteria

The study included 318 nursing professors in nursing at federal and state public universities in Brazil with an undergraduate nursing course, with no restrictions regarding the presence or absence of a graduate degree in the area. Effective teaching nurses in nursing at a public university in Brazil and working remotely, hybridly or in person were included. Professionals with no nursing degree, on a temporary contract at the institution, on leave of any nature, on leave for professional qualification or teaching at a private university were excluded.

### Study protocol

The 3D-WFI^(2)^ was developed among US workers using a national pilot study (n= 204) and a national validity study (n= 2,477). The 18-item measure assesses how often a person is exposed to workplace fatigue, ranging from never experiencing workplace fatigue, through infrequent exposure (less than once a month), to frequent exposure (every day). It provides proportional assessments of physical (six items), mental (six items), and emotional (six items) workplace fatigue. The scale is a five-point Likert type (1 every day; 2 - at least once a week; 3 - at least once a month; 4 - less than once a month; and 5 - never).

Before starting the study, the consent of the main authors of the instrument^(2)^ was requested via e-mail to carry out the translation and cross-cultural adaptation, following international recommendations^(8)^. To this end, the original version of the 3D-WFI inventory was translated from English into Brazilian Portuguese by two bilingual translators, independently: one of them had experience with translations related to the health area; and the other had no knowledge about the translation objectives and had no experience with translations related to the health area. A third person assessed the entire translation and back-translation process and proposed a final version of the instrument.

An expert committee, composed of five experts in the field of health with experience in workers’ health, assessed semantic, cultural, idiomatic and conceptual equivalences as well as the face validity of each scale, approving them to be used in pre-test in order to develop the pre-final version of the instrument. Furthermore, the Content Validity Index (CVI) and the concordance rate (CR) were calculated for each translated item. The calculations referring to the assessment of the translated scale obtained 1.00 of CVI. Regarding the 3D-WFI inventory feasibility, all items had an CR of 100%.

In the pre-test phase, the 3D-WFI version, validated by the expert committee, was applied individually to 30 nurses, students of master’s or doctoral courses in nursing, who had already developed some activity as a professor. Each participant was given the opportunity to contribute to the research by reporting their difficulties and facilities in completing it and leaving suggestions, when necessary. At the end of the phase, there was no need to change the instrument.

After the cultural adaptation procedures of the instruments, the final version of 3D-WFI-Brazil was considered approved for application in the selected sample for construct validity and obtaining its psychometric results. The sampling method used was non-probabilistic of the convenience type. For participant characterization, a questionnaire was used, contemplating characterization variables, variables about professional trajectory and variables about work. The questionnaire also included the 3D-WFI instrument.

Participants were recruited individually. Invitations were sent via e-mail to the faculty. The content of the email described the purpose of this study, its objectives, link to access the instrument, instructions and deadline for completing the questionnaire. In addition to this, so that each participant remembered to participate in the study, five contact attempts were made by email throughout the research period. Moreover, invitations were also sent through social media (Facebook^®^ and Instagram^®^) and WhatsApp^®^ groups. Also, as promotional material, a 46-second video was used and an invitation art with access to the link to the form. Regarding the time to complete the questionnaire, an average time of 17 minutes was estimated in the pre-test phase.

### Analysis of results, and statistics

The assessment of 3D-WFI’s psychometric properties was carried out through a cross-sectional study with a sample of 318 nursing professors from public universities in Brazil.

To calculate the sample size, we considered the total estimated value of 1,525 professors from federal and state public universities with an undergraduate nursing course. The calculation was performed using the EpiInfo program, version 7.2, using a 95% confidence level, obtaining a minimum sample of 307 participants. Data were recorded in a virtual environment and answered automatically on Google Forms, being organized, exported and tabulated later using Microsoft Office Excel^®^.

For statistical analysis, data underwent an extensive and robust process of testing the properties, with a combination of exploratory factor analysis (EFA) techniques, performed in the FACTOR 12.01.02 software, and confirmatory factor analysis (CFA), performed in the JASP software 0.16.1.0, aiming to seek strong evidence of validity in the construct stage and its stability for other subsamples. EFA requires the fulfillment of several steps, such as data inspection techniques, factor analysis method, retention and rotation technique, and factor loading quality indices^(9)^.

Dimensionality in EFA was tested by parallel analysis (PA), which has been considered one of the most effective and accurate techniques for testing the number of factors/dimensionality^(10-11)^. Test robustness was determined from the association of a bootstrap with a sample extrapolation to 5,000. Polychoric matrix estimation was performed using the Bayes Modal Estimation^(12)^.

Analysis was implemented using a polychoric matrix and Robust Diagonally Weighted Least Squares (RDWLS) extraction method^(13)^. The decision on the number of factors to be retained was performed using the PA technique with random permutation of the observed data and the rotation used was Robust Promin^(14)^.

The instrument’s quality parameters were estimated by the instrument’s explained variance, which should be around 60%^(15)^, and by the initial factor loadings of 0.30, which are recommended when the sample has less than 300 individuals^(9,16)^, but the model must look for factor loadings above 0.50^(16)^ and the commonalities must have values above 0.40^(9)^.

Also, commonalities must have values above 0.40^(16)^. Keeping or removing an item from the model will depend on the magnitude of commonalities, factor loadings, sample size and the degree to which the item can measure the factor and the lack of cross-loading^(9)^.

As a complementary analysis, factor stability was assessed using the H Index (Ferrando & Lorenzo-Seva, 2018), using the Robust Promin technique (h and w) (robust rotation)^(15)^, which assesses the importance of items for instrument structure. The variable with the least stable set of correlations (that is, a large value of h) will have a weight value (w) close to zero. On the other hand, a variable with a set of perfectly stable correlations will have a weight value (w) of 1. The higher the value in w, the more important the variable is to define the simple structure of the factorial solution, i.e., the more important is the item for the instrument^(15)^.

The G-H index assesses how well a set of items represents a common factor. H values range from 0 to 1. High H values (> 0.80) suggest a well-defined latent variable, i.e., stable across different studies. Meanwhile, low H values suggest an ill-defined and probably unstable latent variable across different studies^(17)^.

Still, for the factor estimation quality and effectiveness, Factor Determinacy Index (FDI), pointing to an adequate estimate of values greater than 0.90, Overall Reliability of fully-Informative prior Oblique N-EAP scores (ORION) marginal reliability (> 0.80), sensibility ratio (SR) (> 2) and expected percentage of true differences (EPTD) (> 90%) were used^(17)^. The application of multiple indicators is justified due to the need to attest, through various techniques, the instrument’s validity evidence. Moreover, the application and interpretation of goodness-of-fit (GOF) model adjustment indices do not guarantee that factor analysis solution is good or useful in practice, as it is possible to obtain satisfactory solution indices based on low quality items^(17-18)^.

Instrument reliability was assessed using Cronbach’s alpha, McDonald’s omega and composite reliability (CR) indicators. Both reliability indicators are considered acceptable with values ≥ 0.70^(19)^. The adoption of three indicators sought to increase interpretation reliability, as numerous inconsistencies in reliability have been reported using Cronbach’s alpha^(20-21)^.

For adjustment indices in CFA, factor loadings greater than 0.50 and the following minimum indices for adequacy were considered, considering the number of participants and variables through adjustment indices as follows: Non-Normed Fit Index (NNFI); Comparative Fit Index (CFI); Goodness Fit Index (GFI); Adjusted Goodness Fit Index (AGFI); Root Mean Square Error of Approximation (RMSEA); and Root Mean Square of Residuals (RMSR). According to the literature^(9)^, NNFI, CFI, GFI and AGFI values must be above 0.95, and RMSEA and RMSR values must be less than 0.08^(9)^.

## RESULTS

Among the 318 participating teaching nurses, 87.7% were female, aged between 25 and 68 years (42 ± 9.4); 252 (79.2%) were affiliated with federal universities; and 66 (20.8%) were linked to state universities. The average time since graduation in higher education was 18 years (± 9.4). In addition to the activities carried out at the undergraduate level, 88 (27.7%) nursing professors also develop activities at the graduate level (*Lato Sensu* and *Scrito Sensu*). Missing value was not identified in the model.

To ensure that the data fit a polychoric correlation analysis model, they were subjected to the Kaiser-Meyer-Olkin test (KMO= 0.95) and Bartlett’s sphericity test (3570.9; gl=153; p=0.000), which indicated good levels of factorability^(22)^.

For the decision to remove or keep the items in the instrument, factor loadings, commonalities and sample size were assessed. This study interviewed 318 individuals, which is considered by Sellbom and Tellegen (2019) to be a median sample^(16)^. Factor loadings were established between 0.50 and 1.0 in the sample, indicating satisfactory and adequate levels^(16)^. The commonalities were above 0.40, with a variation between 0.40 and 0.82, showing that the items measure the latent variable.

Items 10 (“*Quis se desligar mentalmente do trabalho ao final do dia*?”) and 15 (“*Se sentiu emocionalmente exausto (fatigado/gasto*”) had factor loadings equal to 1, the which made it necessary to check item residual variance^(23-24)^. When checking the residual variance, it was decided to keep the two items in the model, since the residual variance was not negative (0.61 and 0.95, respectively). Factor loadings equal to or greater than 1 may also indicate estimation error, called Heywood Case, and the most common is when there are similar items in the measure, causing what is called a linear combination of variables, where other instrument variables explain 100% of that item’s variance^(17,25)^.

Although items 10 and 15 are semantically similar, it was decided to keep the items in the model, as they belong to different dimensions (item 10: mental fatigue and item 15: emotional fatigue), i.e., they are intended to verify a specific moment^(26)^. In this regard, it is also possible to think that perhaps these items may be showing interference related to cross-cultural adaptation with regard to idiomatic, semantic or even cultural aspects, or may even denote the possibilities of interpretation that the statement allows. Moreover, CFA showed that the factor loadings of the two items were > 0.5 (0.77 and 0.84, respectively) as recommended in the literature^(9)^, confirming that there is no redundancy between items and the importance from items to construct. Thus, the 3D-WFI was adapted to Brazilian Portuguese (*Inventário de Fadiga Ocupacional em Três Dimensões* - (3D-WFI)), maintaining the structure with 18 items and three factors, with 62.77% variance.

To obtain greater accuracy and quality in the validity technique of the instrument and bring more information to the model, the robust rotation technique was used (h and w)^(15)^. Table 1 shows the corresponding robust breasts (e). As can be seen, the item that produced the least stable set of correlations (Item 2 “*Teve dificuldade em se envolver em atividade física ao final do dia de trabalho?*”) received a weight of 0.00, while the item that produced the most stable set of correlations (Item 17 “*Se sentiu emocionalmente esgotado (sem energia) ao final do dia de trabalho?*”) received a weight of 0.48. Table 1 shows the values of factor loadings, residual variance, commonalities and robust rotation (h and w).

**Table 1:** Factor loadings, commonalities and robust rotation (h and w) of the Brazilian version of 3D-WFI (N= 318), Brazil, 2021

Item	Factor loadings			Residual variance	Communalities	Robust rotation	
	F1	F2	F3			h	W
*Fadiga Física*							
*1. Se sentiu fisicamente cansado ao final do dia de trabalho?*	0.13	-0.15	0.67	0.32	0.45	0.85	0.33
*2. Teve dificuldade em se envolver em atividade física ao final do dia de trabalho?*	0.05	-0.18	0.60	0.87	0.72	1.28	0.00
*3. Se sentiu fisicamente exausto (fatigado/gasto fisicamente) ao final do dia de trabalho?*	-0.10	0.03	0.95	0.93	0.79	0.73	0.43
*4. Quis se afastar fisicamente do trabalho ao final do dia?*	0.32	0.09	0.25	1.19	0.40	0.82	0.35
*5. Se sentiu fisicamente esgotado (sem energia) ao final do dia de trabalho?*	0.21	0.04	0.64	0.69	0.75	0.69	0.45
*6. Quis evitar qualquer situação que exigisse muita energia física ao final do dia de trabalho?*	0.64	-0.04	0.18	0.88	0.58	0.86	0.32
*Fadiga Mental*							
*7. Se sentiu mentalmente cansado ao final do dia de trabalho?*	0.71	-0.01	0.12	0.49	0.65	0.67	0.47
*8. Teve dificuldade para pensar e se concentrar ao final do dia de trabalho?*	0.82	0.03	-0.11	0.73	0.57	0.89	0.30
*9. Se sentiu mentalmente exausto (fatigado/gasto mentalmente) ao final do dia de trabalho?*	0.50	0.12	0.26	0.64	0.73	0.69	0.46
*10. Quis se desligar mentalmente do trabalho ao final do dia?*	1.0	-0.07	-0.19	0.61	0.69	0.82	0.35
*11. Se sentiu mentalmente esgotado (sem energia) ao final do dia de trabalho?*	0.83	-0.02	0.05	0.66	0.81	0.71	0.44
*12. Quis evitar qualquer situação que exigisse muita energia mental ao final do dia de trabalho?*	0.87	-0.01	-0.00	0.74	0.73	0.72	0.43
*Fadiga Emocional*							
*13. Se sentiu emocionalmente cansado ao final do dia de trabalho?*	0.26	0.71	-0.09	0.75	0.74	0.69	0.45
*14. Teve dificuldade para demonstrar ou lidar com emoções ao final do dia de trabalho?*	-0.06	0.95	-0.13	0.98	0.65	0.92	0.28
*15. Se sentiu emocionalmente exausto (fatigado/gasto emocionalmente) ao final do dia de trabalho?*	-0.18	1.05	0.00	0.95	0.82	0.87	0.32
*16. Quis se desligar emocionalmente do trabalho ao final do dia?*	0.27	0.67	-0.12	1.05	0.65	0.73	0.43
*17. Se sentiu emocionalmente esgotado (sem energia) ao final do dia de trabalho?*	-0.13	0.85	0.19	0.96	0.81	0.66	0.48
*18. Quis evitar qualquer situação que exigisse muita energia emocional ao final do dia de trabalho?*	0.01	0.75	0.09	1.07	0.70	0.73	0.43

Item reliability was estimated using Cronbach’s alpha (α) and McDonald’s omega (ω), resulting in 0.95 and 0.97, respectively. Construct replicability was assessed by the Generalized G-H Index with an index greater than 0.80. One can infer a stability of the model even when applied to other population samples and its consequent generalization. In addition, factor CR was also adequate (>0.70)^(17)^.

For the factor estimation quality and effectiveness, FDI, ORION, SR and EPTD were used. They also indicated a quality and effectiveness of the model^(17)^. Table 2 presents the results of quality indicators and estimated scores.

**Table 2:** Quality indicators and score estimation of the Brazilian version of the 3D-WFI instrument (N=318), Brazil, 2021

Indicators	PF	MF	EF
G-H Index	0.95	0.96	0.92
Composite reliability	0.91	0.93	0.81
Factor Determinacy Index	0.97	0.97	0.95
ORION Marginal Reliability	0.95	0.95	0.91
Sensitivity ratio	4.40	4.45	3.30
Expected percentage of true differences	95.4%	95.6%	93.3%

Note: PF - Physical Fatigue; MF - Mental Fatigue; EF - Emotional Fatigue.

CFA presented factor loadings ranging from 0.50 to 0.90. The model’s quality indices were established at: χ2= 35.590; gl=132; NNFI=1.0; CFI=1.0; GFI=0.99; AGFI=0.99; RMSEA=0.00; and RMSR= 0.00. Parsimonious fit measures were χ^2^/gl= 0.26, and PGFI, 0.85. The results of confirmatory analysis showed that the Brazilian version of 3D-WFI consists of three-dimensional structures including mental, physical and emotional fatigue, similar to the factorial structure obtained in the original study^(1)^.

**Figure 1:**
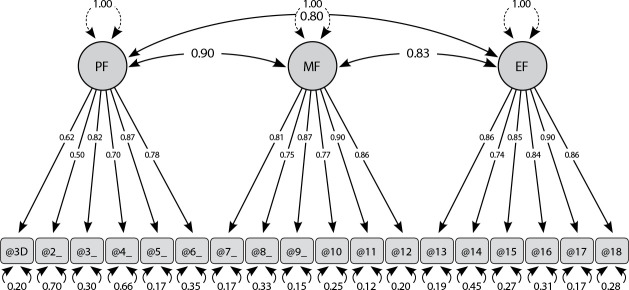
Path diagram of the Brazilian version of 3D-WFI

*Note: PF- Physical Fatigue; MF- Mental Fatigue; EF - Emotional Fatigue.*

The correlations (R) between the 3D-WFI version dimensions were established between 0.80 and 0.90, ranging from moderate to strong. The physical fatigue dimension showed a strong correlation (r= 0.90) with the mental fatigue dimension. The physical fatigue dimension, when correlated with the emotional fatigue dimension, showed a moderate correlation (r= 0.80). The same happened in the correlation between the mental fatigue dimension and the emotional fatigue dimension (r= 0.83). In Figure 1, path diagram, the results of factor loadings, correlation between dimensions (R) and standard error are presented.

## DISCUSSION

Workplace fatigue is seen as a personal and work-related condition that is integrated into the health, attitude, safety and performance of workers. Work fatigue is associated with extreme exhaustion and tiredness with a decrease in work capacity that is felt during and at the end of workdays^(2)^. Therefore, an instrument for its measurement is necessary, with 3D-WFI being the most used^(1,4,27-28)^.

The process of adapting and validating the 3D-WFI followed the steps suggested by the literature^(8)^, which involved translation, synthesis of translations, backtranslation and semantic and content validity with the target population and with judges, respectively, and pre-test. Content validity was carried out by the expert committee that contributed to semantic, idiomatic, conceptual and experiential assessment. The adequacy of the Portuguese version to the construct was evidenced by experts’ agreement, considering that the minimum agreement of 80% among judges is a decision criterion on item pertinence to the factor that theoretically refers to^(26)^.

For psychometric validity studies, the literature recommends sampling over 50 individuals, with a minimum of 100 people, to guarantee solid conclusions, based on an average of 5 or more observations per item^(9)^. The present study interviewed 318 teaching nurses, which ensured an average of 15.9 observations for each item of 3D-WFI.

The results of this study demonstrated that the Brazilian version of 3D-WFI presented a three-dimensional characteristic, satisfactory factor loadings and good levels of reliability for all techniques (EFA and CFA) and indicators used in them, which point to an instrument with evidence of a consistent and reliable internal structure for measuring the desired construct. Although the study does not have a comprehensive sampling process in order to configure the representative of nursing professors at federal and state public universities in Brazil, it was found that the instrument has good psychometric properties for measuring the construct of workplace fatigue in Brazilian adults. The validity explored with EFA allowed the permanence of all items in the 3D-WFI instrument.

In terms of reliability, 3D-WFI items showed high values for both criteria (α=0.95 and ω = 0.97). Furthermore, RC indicated reliability, with results greater than 0.70 (0.91, 0.93 and 0.81). Although alpha is not a good indicator for comparing the models, as has been reported in the current literature^(20-21)^, it was the only common indicator between our study and the others that tested the 3D-WFI. The α values were the same as those found in the original study carried out with workers who performed different occupations in industries in the United States (α = 0.95)^(2)^ as well as in other populations such as Lebanese physicians and medical students (α = 0.95)^(4)^. Lower values of α for the instrument were verified in other studies with Lebanese pharmaceutical workers (α = 0.88) and German workers (α = 0.77)^(1,28)^. It was not possible to make comparisons of RC findings with other 3D-WFI validity studies. Furthermore, the G-H index indicated replicability of the model in other populations and subsamples^(17)^.

Commonalities were above 0.40, with a variation between 0.40 and 0.82, showing that the items measure the latent variable and the factor loadings, most items factored above 0.5, as recommended by the literature^(16)^. Although items 10 (“*Quis se desligar mentalmente do trabalho ao final do dia?*”) and 15 (“*Se sentiu emocionalmente exausto (fatigado/gasto emocionalmente) ao final do dia de trabalho?*”) presented factorials equal to 1, it was decided to remain in the instrument.

Regarding the instrument’s quality parameters, the explained variance of the instrument should be around 60%^(14)^. Our model had an explained variance of 62.77%, as recommended in the literature.

To obtain greater accuracy and quality in the instrument’s validity technique and bring more information to the model, the robust rotation technique (h and w) was used. The higher the value of w, the more important the variable for the instrument^(15)^.

Thus, the most important items for the instrument in each dimension was item 3 (“*Se sentiu fisicamente exausto (fatigado/gasto fisicamente) ao final do dia de trabalho?*”) (w= 0,43) and item 5 (“*Se sentiu fisicamente esgotado (sem energia) ao final do dia de trabalho?*”) (w= 0.45) in the physical fatigue dimension; item 7 (“*Se sentiu mentalmente cansado ao final do dia de trabalho?*”) (w= 0.47) and item 9 (“*Se sentiu mentalmente exausto (fatigado/gasto mentalmente) ao final do dia de trabalho?*”) (w= 0.46), in the mental fatigue dimension; item 13 (“*Se sentiu emocionalmente cansado ao final do dia de trabalho?*”) (w= 0.45) and item 17 (“*Se sentiu emocionalmente esgotado (sem energia) ao final do dia de trabalho?*”) (w= 0.48), in the emotional fatigue dimension. None of the items received a weight close to 1, as they all produced correlations with a considerable amount of sampling error, as recommended by the literature^(15)^.

In CFA, the factor loadings of 3D-WFI items were greater than 0.50 (0.50 to 0.90), and fit indices (RMSEA= 0.00; CFI= 1.0; χ2= 35.590) showed closer than the literature suggests^(9)^. Our results are close to the Arabic^(4)^ and German^(1)^ versions of 3D-WFI. In the Arabic version, factor loadings ranged between 0.86 and 0.91, fit indices were adequate (RMSEA = 0.05; CFI =0.98; χ2 = 295.76)^(4)^. Adjustment indices were also adequate in the German version (RMSEA=0.06; CFI=0.96; χ2= 356.00) and factor loadings ranged from 0.71 to 0.93^(1)^. These findings endorse the instrument quality, because when consistency proves to be reliable in repeated measurement processes, it corroborates its operational potential for use in other population studies^(26)^.

The results of confirmatory analysis showed that the 3D-WFI consists of three-dimensional structures including mental, physical and emotional fatigue, similar to the factorial structure obtained in the original study^(2)^ and version in other languages^(1,4-7)^. Furthermore, the correlation coefficients between the three factors were also adequate.

The correlations (R) between the 3D-WFI version dimensions were established between 0.80 and 0.90, ranging from moderate to strong. From a theoretical point of view and external validity, it was found that all dimensions of fatigue were highly related to each other, indicating that fatigue is an element of tiredness and loss of energy that retains common and extensive aspects between cognitive, emotional and physical domains^(2)^.

The highest correlation between the physical fatigue and mental fatigue dimensions stands out (r= 0.90). Similar results were found in the Arabic study (r= 0.70)^(4)^. Reduced functional capacity reflects a decrease in the ability and/or motivation to respond to certain stimuli or engage in certain types of activities. Meanwhile, mental fatigue can be felt after or during prolonged periods of cognitive activity and involves tiredness or even exhaustion, an unwillingness to continue with the current activity, and a decreased level of commitment to work^(2)^. These results highlight the impact that fatigue can have on workers’ health, but mainly on mental health, which can become a serious public health problem^(1)^.

### Study limitations

Study limitations are mainly related to the representativeness of the studied sample, since it included a portion of teaching nurses. Thus, it is recommended that future studies expand the application of this instrument, aiming to understand workplace fatigue in other samples and populations.

### Contributions to nursing, health, or public policies

Two contributions stand out: first, reflection on the possibility of investing in workers’ health care in order to maintain or improve individuals’ responses to workplace fatigue. Consequently, it can produce health, or minimize the effects of workplace fatigue. Second, the psychometrics methodological framework, which comes from epidemiological studies and psychology, contributing to the construction of knowledge in several areas of nursing practice.

## CONCLUSION

The statistical procedures carried out led to the conclusion that the *Inventário de Fadiga Ocupacional em Três Dimensões* (3D-WFI) is a reliable, valid instrument with solid psychometric properties for assessing fatigue among Brazilian workers. Collectively, the original study and the present study support the importance of differentiating between physical, mental, and emotional fatigue from work, to develop a more complete and integrative understanding of the experience, causes, and results of fatigue while working. Furthermore, this instrument can also help in the development of both individual and collective actions and strategies to promote workers’ health.
